# Three patterns for identifying the posterior plane of a lenticule during SMILE

**DOI:** 10.1007/s10792-023-02760-9

**Published:** 2023-06-24

**Authors:** Ke Zheng, Jing Wang, Xiaohong Zheng, Yinan Han, Xingtao Zhou

**Affiliations:** 1 NHC Key Laboratory of Myopia, Fudan University, Shanghai, China; 2 Key Laboratory of Myopia, Chinese Academy of Medical Sciences, Beijing, China; 3 Eye Institute and Department of Ophthalmology, Eye and ENT Hospital of Fudan University, Shanghai, 200031 China; 4 Shanghai Research Center of Ophthalmology and Optometry, Shanghai, China; 5 Department of Ophthalmology and Vision Science, Eye and ENT Hospital of Fudan University, Shanghai, China

**Keywords:** Small-incision lenticule extraction, Lenticule dissection, Double lines, Leaf sign, Triple lines

## Abstract

**Purpose:**

To describe three patterns of posterior plane edge identification in small-incision lenticule extraction and to prevent lenticule mis-dissection.

**Methods:**

Femtosecond laser application was performed for small-incision lenticule extraction (SMILE) by one surgeon. The surgical videos of SMILE were recorded and re-watched by the surgeon after operation.

**Results:**

Small-incision lenticule extraction was performed in 52 eyes of 28 patients, and no patient had cap-lenticular adhesion. Three patterns of posterior plane of lenticule were noticed when the surgical videos were re-watched. A “double lines” attached to the dissector were visible, signifying the reflective tape of the edge of the lenticule and the cap. During the expansion of the posterior lamellar separation, a fusiform opening between the lenticule edge and the underlying matrix layer was assumed to be a “leaf sign.” With some unintentional operation, the posterior lamella was pushed away from the surgeon. The edge of the lenticule away from the anatomical part, the marking of the femtosecond laser cut, and the edge of the cap layer showed three reflective bands, which formed a “triple lines.” The “double lines,” “leaf sign,” and “triple lines” were observed in 30 eyes (57.7%), 21 eyes (40.4%), and 1 eye (1.9%), respectively.

**Conclusion:**

These three signs cover possible situations and provide visual landmarks to identify the correct dissection of the posterior plane, which can help shorten the learning curve of novice doctors.

## Introduction

Small incision lenticule extraction (SMILE) is currently the most innovative refractive surgery to treat myopia and astigmatism [1-3]. It combines femtosecond laser technology with high-precision lenticule extraction. SMILE is a flapless, single-step procedure that preserves the structure of the front corneal tissue. The potential advantages of SMILE over other laser refractive surgeries include reduced keratocyte damage, less corneal inflammation and discomfort, and less iatrogenic dry eye [4].

The standard technique for SMILE involves docking, femtosecond laser application, lenticule dissection, and extraction [5, 6]. Lamellar dissection and removal of the lenticule should be manually completed through a 2-mm corneal incision after creating the lenticule within the cornea. Identifying the anatomic lenticule structure and the correct dissection plane is the most critical step for surgeons who are newly acquainted with SMILE. This novel technique could be more technically challenging and associated with a significant learning curve [7-9]. The failure of lenticule dissection can even lead to complications such as tearing and rupture of the corneal cap or lenticule [7, 10, 11]. Most of the complications are avoidable with a clear identification of anterior and posterior planes. We introduce three patterns for identifying the posterior plane of the lenticule during SMILE that may help shorten the learning curve.

## Materials and methods

This study evaluated the eyes of consecutive patients who underwent small-incision lenticule extraction to correct myopia or myopia astigmatism at the Eye and ENT Hospital, Fudan University, China, between March and April 2020. All the patients voluntarily agreed and provided signed informed consent after a detailed explanation of the study. The study adhered to the tenets of the Declaration of Helsinki and was approved by the Ethics Committee of the Eye and ENT Hospital (KJ2010-18), Fudan University, China.

### Patient examination

All patients had routine ophthalmic examinations preoperatively, which included uncorrected distance visual acuity (UDVA), corrected distance visual acuity (CDVA), manifest and cycloplegic refractions, length of axis, the diameter of the scotopic pupil, white-to-white corneal diameter (W-W), intraocular pressure (IOP), corneal thickness, corneal topography, slit-lamp microscopy, and dilated indirect fundoscopy.

### Surgical technique

The VisuMax FS laser system (Carl Zeiss Meditec AG, Berlin, Germany) was used to perform the SMILE procedure; each performed under surface anesthesia by a single experienced surgeon (KZ). The 500-kHz repetition rate, 130-nJ pulse energy, 120-μm cap thickness, 7.5-mm cap diameter, and 6.4- to 6.7-mm lenticule diameter were set as laser parameters according to the diameter of the scotopic pupil and manifest refraction. A side cut of 2 mm at the superior 12-o’clock position was created in all patients, with spot and track spacing of 4.5 μm for the cap bed, 2.5 for the cap side-cut, and 2.0 μm for the lenticule side-cut. When femtosecond laser application was completed, the patient bed was moved to the observation position beneath the SMILE microscope. The anterior plane was dissected successfully before posterior plane dissection in all eyes. The process of SMILE was recorded as videos and re-watched by the surgeon after operation.

## Results

**Fig. 1:**
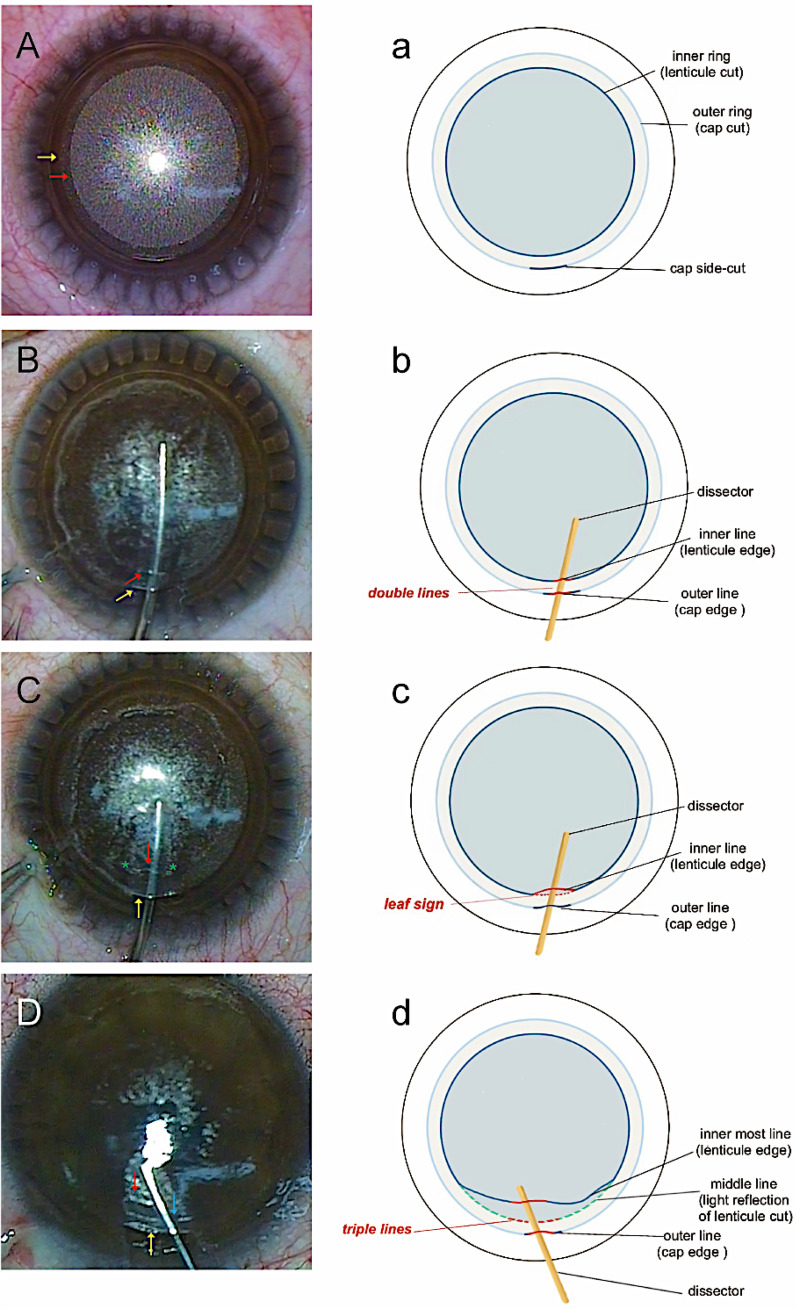
Three patterns of the posterior plane of the lenticule in small-incision lenticule extraction. **A** The cap layer (which has low density of bubbles) and the uniform bubble layer (the lenticular layer, which has high density of bubbles) formed concentric circles after femtosecond laser application was completed. The outer ring (yellow arrow) corresponded to the cap cut and the inner ring (red arrow) corresponded to the lenticule cut. **B** “Double lines” was observed when the edge of lenticule and cap were attached to the dissector. The outer line (yellow arrow) corresponded to the cap edge and the inner line (red arrow) corresponded to the lenticule edge. **C** “Leaf sign” was observed when the lenticule edge was slightly pushed up. The edge of lenticule (red arrow), the matrix below and their two connecting points (green *) formed a leaf-shaped opening. **D** “Triple lines” was observed when part of the lenticule near the side-cut was dissected and pushed away from the original anatomical position. The outermost line (yellow arrow) corresponded to the cap edge and the innermost line (red arrow) corresponded to the lenticule edge. And the line in the middle (blue arrow) was the light reflection of the trace after lenticule cut. It was a pseudo-line. a, b, c, and d are reduced graphs that aids in understanding A, B, C, and D, respectively

Three patterns of the posterior plane of the lenticule were generalized into three signs: “double lines,” “leaf sign,” and “triple lines,” which are detailed in the following paragraphs. The uniform bubble layer and the cap layer were clear before dissection in all eyes (Fig. 1A, a). A total of 52 eyes of 28 patients were included in the analysis, and no patients had cap-lenticular adhesion. The “double lines,” “leaf sign,” and “triple lines” were observed in 30 eyes (57.7%), 21 eyes (40.4%), and 1 eye (1.9%), respectively.

### Double lines

When creating the channel into the posterior plane after the complete dissection of the anterior lamella or delineating the posterior lamella in advance through the side cut, two reflective lines can be observed on the dissector. The inner line reflects the edge of the posterior lamella (lenticule), while outside line reflects the anterior lamella (cap). The reflections of the two lines near the incision are called “double lines” (Fig. 1B, b).

### Leaf sign

In some cases, during the creation of the posterior lamellar channel, the lenticule edge is slightly pushed inward, and a small “leaf-shaped” opening appears at the side-cut. Except for the edge, the posterior lamella is still attached to the underlying matrix, and the two connecting points of the lenticule and matrix are visible on both ends of the leaf (Fig. 1C, c). The dissector can slide into the “leaf-shaped” gap, confirming the correct plane of lenticule dissection, and then, the dissection can be completed smoothly.

### Triple lines

Sometimes the lenticule can be dissected and pushed away from the original anatomical position due to unintentional intraoperative manipulation so that the edge of the lenticule has a certain distance from the trail of the lenticule circumference. The connecting points of the lenticule and underlying matrix mentioned in the “leaf sign” are invisible. When confronted with this situation, the judgment of the posterior plane may be difficult for a novice surgeon even with successful cap dissection. The dissector is slowly brought forward again, reaching the edge of the lenticule that has been pushed and pleated away from the surgeon. Three parallel reflective stripes are visible. The farthest reflective stripe from the surgeon is the reflection of the lenticule edge. Actually, the reflective band in the middle is the femtosecond laser scan trace of the lenticule rim, which is beneath the dissector. The outermost reflective band is the reflection of the cap edge, which is hanging on the dissector. These three parallel, concentric stripes of reflection can be summarized as “triple lines” (Fig. 1D, d).

## Discussion

During the entire process of SMILE, dissection of the femtosecond laser-cut lamellar planes is performed manually. How to identify and dissect the anterior and posterior planes successfully is the most important step for the surgeon to accumulate experience. Consensuses on SMILE established by experts in China and other countries [12] suggest that lenticule dissection should be performed via the sequential dissection of anterior interface (under the corneal cap) and then posterior interface of the lenticule. Pre-separating a small portion of the lens and cap near the side cut or pushing the dissector in the area between lenticule and cap, which is 100 percent upper level, helps novices to successfully complete the lenticule separation. A slight upward force of the dissector when separating the upper layer, and a slight downward force when separating the lower layer are also recommended.

Although unintended initial dissection of the posterior plane (UIDPP) is acceptable and remediable when the surgeon is experienced enough to re-separate the upper layer from the opposite direction of the side-cut entry, and the occurrence of UIDPP will not affect the recovery of long-term visual acuity after surgery [13]. For novice, because the lenticule adheres to the corneal cap when UIDPP happens [14], it is then challenging to continue to dissect the lenticule for less experienced doctors, even lead to surgical failure. We summarized the five signs of UIDPP during SMILE in the early stage [15], which can help identify mis-dissections and shorten the learning curve. Also, the use of anterior segment optical coherence tomography (AS-OCT) can help identify separated lenticular planes in difficult SMILE procedures [16]. However, the incidence of unintended initial dissection of the posterior plane is relatively low [17], and identifying the lenticule lamella accurately and quickly after routine dissection of the anterior plane is more meaningful for promotion. We summarize three possible lenticule morphologies to help the novice surgeon dissect and extract the lenticule successfully.

“Double lines” is the most common sign with a clear anatomical structure and is relatively easy to identify [15, 18]. After the femtosecond laser application is completed, a uniform bubble layer, which is actually the lenticule lamellar plane, can be observed in the corneal stroma layer. The light reflection of the lenticule and cap forms a “double lines” sign. By recognizing the double-line sign and experiencing the resistance of the dissector, the gap between the lenticule and the residual matrix bed can be entered effortlessly, and the posterior plane can be dissected successfully.

The appearance of the “leaf sign,” which has been named the “meniscus sign” [19], is also relatively common and easy to identify. The lenticule edge is arched slightly to form a small leaf-shaped opening with two connection points at both ends of the “leaf.” The formation of the “leaf” may be related to the thickness and strength of the dissector and the distance of the dissector entering the posterior lamellar channel. Pushing the dissector forward through this leaf-shaped opening against the resistance between the lenticule and the matrix below makes the dissection of the lenticule successful.

For novice surgeons, the “triple lines” is the most challenging sign for recognition. The plane where the reflective strip in the middle is located in, it belongs neither to the cap nor to the lenticule. It is the corneal stroma plane, and the reflective strip is the impression of the annulus cut on the stroma plane after the femtosecond laser application. It often occurs when an excessive tear enters in the interlayer, blurring the anatomy of the cap, the lenticule and the matrix below. For inexperienced surgeons, improper manipulation and infiltration of tears may cause forward displacement of the lenticule without the “double lines” or “leaf sign.” When three stripes of light reflection are found, it should be noted that the stripe in the middle is a pseudo-stripe, which is the trace of the lenticule edge cut by a femtosecond laser. The middle light reflection stripe must be located under the dissector. Without the understanding of “triple lines,” an inexperienced surgeon may focus on the original anatomical location of the lenticule and cap edge, ignoring the farthest reflective strip from him. The surgeon may have difficulty identifying the posterior plane in this situation. A newly qualified surgeon may even suffer from self-doubt over whether an unintended initial dissection of the posterior plane has been completed and dissect the lenticule in the cap plane.

## Conclusion

For surgeons who are newly acquainted with SMILE, it is advised to delineate and create the anterior and posterior lamellar channels through the side cut. For experienced surgeons, an anterior lamellar channel is advised to create a left-hand position between the “double lines” (for right-handed surgeons). The methods mentioned above can effectively avoid unintended initial dissection of the posterior plane. Excessive tears should be avoided penetrating into interlamination. After successful anterior plane dissection, surgeons can patiently identify the three patterns of the posterior plane. When a “double lines” or “leaf sign” is invisible while a reflective strip is visible below the dissector, surgeons should be alert to the possibility of a “triple lines” if the lenticule cannot be reached. Identifying these three patterns of the posterior plane through the side cut can help surgeons achieve successful separation.

## Data Availability

The datasets used and/or analyzed during the current study are available from the corresponding author on reasonable request.

## Abbreviations

SMILE: Small incision lenticule extraction
UDVA: Uncorrected distance visual acuity
CDVA: Corrected distance visual acuity
W–W: White-to-white corneal diameter
IOP: Intraocular pressure
UIDPP: Unintended initial dissection of the posterior plane
